# A Prospective Study to Assess the Outcome of Motivational Interviewing Among Male Students of Haryana, India: A Strive Towards Smoking Cessation in the Youth

**DOI:** 10.7759/cureus.22642

**Published:** 2022-02-26

**Authors:** Virinder S Gill, Neha Chaudhary, Avneet Randhawa, Manisha Verma, Gurleen K Rai, Shradha Mishra

**Affiliations:** 1 Community Medicine, Gian Sagar Medical College and Hospital, Rajpura, IND; 2 Community and Family Medicine, All India Institute of Medical Sciences, Patna, IND; 3 Community and Family Medicine, Government Medical College Patiala, Patiala, IND; 4 Community Medicine, Government Medical College Patiala, Patiala, IND; 5 Community and Family Medicine, Baba Raghav Das Medical College, Gorakhpur, IND

**Keywords:** nicotine dependence, contemplation ladder, smoking cessation, motivational interview, smokers

## Abstract

Background

The brown plague is a classic example of the modern-day epidemic.Motivational interviewing has been found to increase smokers' readiness to quit, attempts to quit, and reduce smoking levels.Thus, this study, attempts to find out the prevalence of smoking and assess the impact of motivational interviewing on male smoker students (18-30 years).

Methodology

The study was conducted among the male students of educational institutes in Maharishi Markandeshwar University in Haryana. A cross-sectional study to estimate the prevalence of smoking was carried out. With motivational interviewing of the smokers a prospective cohort study was conducted following the smokers for six months. The probability proportionate to size (PPS) sampling method was applied to recruit 830 participants in the study. A self-designed, semi-structured proforma was used to collect data on smoking behavior, level of dependence, and level of motivation to quit. A modified Fagerstrom questionnaire was used to assess the nicotine dependence level. The motivation to quit smoking was measured by the 10 point scale of Contemplation Ladder, Prochaska, and DiClemente transtheoretical model was used to categorize smokers into stages of readiness to change. Statistical analysis was done using SPSS version 16.0 (IBM Inc., Armonk, New York).

Results

The prevalence of smoking was 20.4%. Following motivational interview on the first contact, more than half of the current smokers (66.2%) had high motivation which further increased to 88.13% on the third visit at six months (p < 0.001). Likewise, at first contact, 47% had low nicotine dependence; this increased to 52.5 % at two weeks, and finally, at six months, 53.4% had low nicotine dependence. But this finding was statistically insignificant (p=0.23). It was noted that 21 (16.5%) smokers out of 127 quit smoking. A high degree of motivation, support from family and friends, and a low degree of nicotine dependence were identified as significant independent predictors for smoking cessation.

Conclusion

A satisfying proportion of smokers could attain a high level of motivation for quitting smoking, but less than one-fourth of the current smokers were able to abstain from smoking at the end of the study period. However, the impact of motivational interviewing was not very promising and calls for multi-pronged approach for discouraging smoking.

## Introduction

The brown plague is a classic example of the modern-day epidemic [1]. Known to be as addictive as cocaine, it is the single most preventable cause of death and disability [2]. According to the WHO, amongst the one billion smokers worldwide, 50% are young people who consume six trillion cigarettes per year [3]. In this regard, clinical tobacco cessation counseling is among the most important and cost-effective preventive services that can be offered in medical practice [4]. Among various methods tried to quit smoking, motivational interviewing is crucial for enhancing motivation for behavior change by guiding patients to explore and resolve their ambivalence in the direction of change [5, 6]. Taking into consideration what the smoker wants, combined with a "client-centered" stance, it utilizes specific methods to increase clients' consideration of the advantages of change [5]. With respect to smoking, motivational interviewing has been found to increase smokers' readiness to quit, attempts to quit, and reduce smoking levels [5].

Thus, this study attempts to find out the prevalence of smoking among male students and also to assess the impact of motivational interviewing on smoking cessation among male smoker students.

## Materials and methods

Research question and study design

A longitudinal follow-up study was conducted to estimate the prevalence of smoking among male students as well as the identified smokers among the recruited study participants were followed up for six months so as to determine the impact of motivational interviewing on smoking cessation among male smoker students.

Study area and study population

The study was conducted on male students belonging to the age group of 18 to 30 years across educational institutes of engineering, medical, law, nursing, hotel management, pharmacy, and dental streams located in Maharishi Markandeshwar (MM) University, Mullana, Ambala, Haryana for a period of six months. The study purpose was explained to the students, and those willing to participate in the study who provided written informed consent were included in the study.

Sample size calculation

The minimum sample size was calculated using the prevalence of smoking from the pilot study (21.3%) at 3% precision and 95% CI to be 822. To account for the non-response rate of 15%, the sample size was calculated to be 830 [7].

Sampling technique

The probability proportionate to size (PPS) sampling technique was applied to recruit students from different streams. The total population was found to be 9781. Out of these, 5756 were male. The contribution of each course in terms of the percentage of the total population was calculated, and a proportionate number of the representative population of male students was selected by simple random sampling. 

Study tool and study procedure

A self-designed, semi-structured proforma was used to collect data on smoking behavior, level of dependence, and level of motivation to quit. Further, a modified Fagerstrom questionnaire [8] was used to assess the nicotine dependence level. The higher the score on this questionnaire, the higher the level of dependence. The current and ever smokers were taken up for motivational interviewing (MI) based on cognitive behavior therapy, to change the behavioral stage according to the guidelines of Miller and Rollnick [9]. The motivational interview consisted of a 15 to 30 min face-to-face person-centered motivational counseling session, including a readiness assessment, a reflection of smoking behavior, motivational tools, and a poster depicting the various outcomes of smoking. The motivation to quit smoking was measured by the 10 point scale of the contemplation ladder [10]. Prochaska and DiClemente transtheoretical model [11] was used to categorize smokers into "stages of readiness to change", which consisted of five stages: the pre-contemplation stage, contemplation stage, preparation stage, action stage, and maintenance stage. The motivational tools used were:

a. Cost calculator (personal saving calculator for calculating cost of smoking)

b. Photographs of tobacco-related diseases as health itinerary

c. Telephone calls /Whatsapp messages

d. Help developing plans to quit by:

i. Setting a quitting date ideally within two weeks

ii. Advising to tell friends/ family about the plan to quit and seeking support

iii. Anticipating challenges, including nicotine withdrawal symptoms

iv. Removing all cigarettes from home/cars

v. Risk charting

Participants were enrolled into a Whatsapp group and were sent awareness-generating motivational messages daily for six months to help with quitting. Trained consultants were available around the clock to support participants who had set a quitting date. Cases requiring referral were referred to the Department of Psychiatry for pharmacologic nicotine replacement therapy. Follow-up interviews lasting 20-20 minutes were held in the second week for the smokers' progress assessment. Those found abstaining were congratulated and encouraged to continue the same. They were also encouraged to discuss the benefits of cessation, including the health benefits, the successes they have had, e.g., duration of abstinence, effective coping strategies, and barriers to cessation, including negative mood, irritability, alcohol, with other smokers. Those found to be still smoking were taken up for an in-depth interview to assess barriers faced and discuss ways to fix the same with the smokers' active involvement. At the end of the six months, participants were assessed for their status of smoking and level of motivation. 

Operational definitions used in the study

Ever smoker: Those who had not smoked/chewed tobacco in the past 30 days preceding the survey but had tried in the past (even once/twice).

Current smoker: Those who had smoked/chewed tobacco products on one or more days in the month preceding the survey.

Nicotine dependence: Those who scored less than four on the modified Fagerstrom were classified as having a low dependence, while those with a score of four to seven were moderately dependent, and those with a score of more than seven were highly dependent.

Degree of motivation: On the basis of the contemplation ladder score, current smokers were divided into three groups. Those who scored eight or above on the contemplation ladder were highly motivated to quit, those who scored five to seven were moderately motivated, while students with a score of less than five were had low motivation.

Statistical analysis

Statistical analysis was done using Statistical Package for the Social Sciences version 21 (IBM Inc., Armonk, New York). The prevalence of smoking was presented as frequency and percentages, and its distribution across different age groups was tested using the Pearson Chi-square test. The outcome of motivation in relation to the degree of motivation and nicotine dependence was analyzed using Fisher's exact test. Continuous data was checked for normal distribution. Further, repeated measure analysis of variance (ANOVA) was applied to test for the statistically significant difference in the contemplation ladder score and number of cigarettes smoked per day across the three successive visits. Multiple logistic regression analysis was applied to identify independent predictors of smoking cessation. The level of significance for all tests was set at p<0.05.

## Results

With a response rate of 97.2%, only 807 students completed the given questionnaire. It was observed that the majority (50.1%) of the population in the study was in the age group of 18 to 20 years. Smokers were classified into current and ever smokers according to the WHO definition.

**Figure 1:**
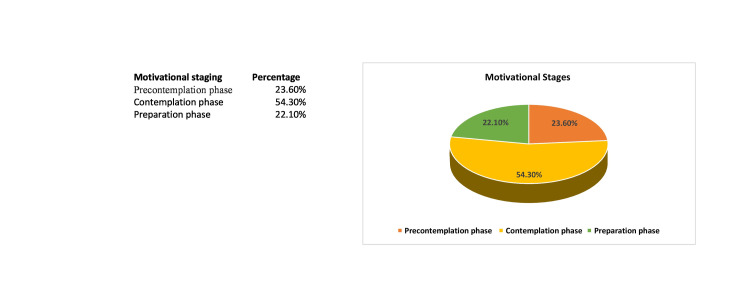
Stages of motivation

The prevalence of smoking was 20.4%, out of which 77% were current smokers, and 23% were "ever smokers". Out of the total current and ever smokers, most subjects in both groups were 18-20 years of age, i.e., 37.8% and 31.6%, respectively. By the age of 20 years, 7.22% of the study population had experimented with smoking. The mean age of current smokers was 22.8 ± 3.5 years. This age variation in smoking was statistically significant (p=0.003) (Table 1). On the basis of the transtheoretical model of Prochasuka and Diclemente for motivation to quit smoking, 23.6% of the current smokers were in the pre-contemplation phase with no intention to quit, 54.3% were in the contemplation phase and considered smoking to be a problem but with ambivalence about the perspective of changing their smoking status and hence had no quitting date planned, 22.1% were in the preparation phase, and none were in the action or maintenance phases (Figure 1). Following the motivational interview at first contact, more than half of the current smokers (66.2%) had high motivation, whereas 33.8% had moderate/low motivation to quit smoking as per the contemplation ladder score. On the second visit at two weeks, only 118 returned for the follow-up, out of which 83.1% had high motivation to quit smoking. Moreover, on the third visit at six months, 88.2% were highly motivated to quit smoking, and only 11.8% had moderate/low motivation to quit smoking. There were nine students who were lost to follow up on successive visits. Likewise, for the ever smokers, on first contact, the majority (94.7%) had a high level of motivation to quit smoking. Further, on the second and third successive visits, a high level of motivation was achieved for all the nonsmokers (100%) (Table 2). Table 3 shows the statistically significant improvement in the contemplation ladder score as well as in the number of cigarettes smoked per day. The mean contemplation ladder score gradually improved on the three successive follow-up visits, i.e., 7.75, 8.30, and 8.74 at first, second, and third contact, respectively (p =0.006). Adding to it, a significant reduction in the mean number of cigarettes smoked per day was observed in the follow-up visits, i.e. 6.12, 4.73, and 4.08 on first, second, and third contact, respectively (p= 0.002).

**Table 1:** Age prevalence of smoking (N= 807) df - degrees of freedom

Age	Current smoker n (%)	Ever smoker n (%)	Nonsmoker n (%)	Test statistics (df) p-value
18 - 20 years	48 (37.8)	12 (31.6)	344 (53.6)	19.6 (6), 0.003 (Pearson's Chi-square test)
21 - 23 years	26 (20.5)	9 (23.7)	115 (17.9)	
24 - 26 years	30 (23.6)	9 (23.7)	119 (18.5)	
27 - 30 years	23 (18.1)	8 (21)	64 (10)	
Total	127 (100)	38 (100)	642 (100)	

**Table 2:** Degree of motivation of current and ever smokers across three visits

Degree of motivation to quit smoking	Current smokers n (%)			Ever smokers n (%)		
	First contact (0 days)	Second contact (two weeks)	Third contact (six months)	First contact (0 days)	Second contact (two weeks)	Third contact (six months)
Moderate/low	43 (33.8)	20 (16.9)	14 (11.8)	2 (5.3)	0 (0)	0 (0)
High	84 (66.2)	98 (83.1)	104 (88.2)	36 (94.7)	38 (100)	38 (100)
Total	127 (100)	118 (100)	118 (100)	38 (100)	38 (100)	38 (100)

**Table 3:** Comparison of contemplation ladder score, number of cigarettes smoked per day, and motivation score across three visits df - degrees of freedom, ANOVA - analysis of variance

Follow-up visit	Contemplation ladder score, mean (SD)	Number of cigarettes smoked per day, mean (SD)
First contact (0 days)	7.75 (2.9)	6.12 (2.1)
Second contact (two weeks)	8.30 (2.5)	4.73 (1.9)
Third contact (six months)	8.74 (2.3)	4.08 (1.2)
Test statistics (df) p-value	34.691 (1.32, 166.5), 0.006 (Repeated measure ANOVA)	32.86 (1.61, 204.03), 0.002 (Repeated measure ANOVA)

Sustained positive outcome of motivational interviewing in the form of quitting smoking was noted for participants who were contracted at first visit. It was noted that 21 (16.5%) smokers out of 127 quit smoking and sustained it through the study period (six months). Out of the total 21 smokers who quit smoking, 85.7% were highly motivated while 14.3% were those whose motivation to quit smoking was low (p<0.001). As far as nicotine dependance is concerned, the majority (76.2%) had low nicotine dependance. Paradoxically, there was not a single individual with high dependence who was able to quit smoking (p=0.008) (Table 4).

**Table 4:** Outcome of motivational intervention among current smokers in relation to the degree of motivation and nicotine dependence (n=127)

Outcome of motivational intervention to quit smoking	Degree of motivation			Total	p-value
	High	Moderate	Low		
Yes	18 (85.7)	0 (0)	3 (14.3)	21 (100%)	<0.001 (Fisher’s exact test)
No	63 (65)	28 (28.9)	6 (6.1)	97 (100%)	
Lost to follow-up	3 (33.3)	0 (0)	6 (66.7)	9 (100%)	
	Degree of nicotine dependence				
	Low	Moderate	High		
Yes	16 (76.2)	5 (23.8)	0 (0)	21 (100%)	0.008 (Fisher’s exact test)
No	41 (42.3)	46 (47.4)	10 (10.3)	97 (100%)	
Lost to follow up	3 (33.3)	3 (33.3)	3 (33.3)	9 (100%)	

Out of the 21 students who quit smoking, 52% were those who started smoking between 17 -19 years, and 33.33% started smoking after the age of 23. Amongst those who started smoking before 16 years of age, only 14% were able to quit smoking. The association between age of initiation and quitting came out to be highly significant (p<0.001) (Table 5). Although, on multiple logistic regression, age at initiation and also the number of cigarettes smoked per day were not identified as independent predictors of smoking cessation. However, a high degree of motivation (OR=3.3), support from family (OR=9.2), and low degree of nicotine dependence (OR=4.6) were identified as significant independent predictors for smoking cessation. The model explained 26.1% variability in smoking cessation (Table 6). 

**Table 5:** Relationship between the age of initiation and quitting among current smokers

Stopped smoking	Age of initiation group					Total
	11-13 years	14-16 years	17-19 years	20-22 years	23-25 years	
Yes	0 (0%)	3 (14.28%)	11 (52.38%)	7 (33.33%)	0 (0%)	21 (100%)
No	2 (2.06%)	17 (17.52%)	41 (42.26%)	34 (35.50%)	3 (3.09%)	97 (100%)
Lost to follow-up	3 (33.33%)	3 (33.33%)	0 (0%)	0 (0%)	3 (33.33%)	9 (100%)
p-value	<0.001 (Fisher’s exact test)					

**Table 6:** Predictors for successful smoking cessation (n=127) AOR - adjusted odds ratio, cpd - cigarettes per day

Variable	Quit smoking		AOR	95% CI	p-value
	Yes n (%)	No n (%)			
Degree of motivation					
Low/moderate	3 (14.3)	40 (37.7)	1	-	-
High	18 (85.7)	66 (62.3)	3.3	1.4-15.1	0.02
Support from family					
Yes	15 (71.4)	20 (18.9)	9.2	2.3-21.4	<0.001
No	6 (28.6)	86 (81.1)	1	-	-
Age of initiation					
Less than 16 years	3 (14.3)	25 (23.6)	1	-	-
More than 16 years	18 (85.7)	81 (76.4)	1.8	0.02-7.9	0.406
Degree of dependence					
High/moderate	5 (23.8)	65 (61.3)	1	-	-
Low	16 (76.2)	41 (38.7)	4.6	2.1-12.3	0.003
Number of cigarettes					
1 to 5 (cpd)	15 (71.4)	60 (56.6)	1.5	0.08-18.7	0.286
>5 (cpd)	6 (28.6)	46 (43.4)	1	-	-
Negelkerke R^2^	0.261				
Hosmer Lemeshow test p-value	0.913				

## Discussion

Tobacco use, primarily cigarette smoking, is the leading cause of preventable morbidity and mortality in the world [12]. Smoking cessation is among the most cost-effective measures in primary care [13]. Despite clear evidence about the harmful effects of smoking, the self-reported prevalence of smoking was 20.4%, and the prevalence of current smoking was found to be 15.73%, which was in line with the WHO report on the tobacco epidemic of 2013 [14]. According to the Global Adult Tobacco Survey (GATS) 2016-2017 data [15], the overall prevalence of smoking was reported to be 10.7% and current smoking to be 12.8 % among males. Studies done by Rani et al. [16] revealed the prevalence of current smoking to be 16%. The prevalence of current smokers in the study was 20.44%, with 4.70% being ever smokers, which was in contrast to a study done by Brar et al. [4], where the current smokers were at 24.3% and ever smokers at 42%. This difference in results should be due to a difference in the study population, wherein Brar et al. [4] studied only the medical students involving more stressful and demanding study courses, while in the present study, representatives from non-medical studies were largely included. Out of the total 127 current smokers, 66% were highly motivated to quit smoking. Similar to our study, Kumar et al. [17] found 15% to be in the pre-contemplation while 85% were in the contemplation stage. Out of 127 current smokers enrolled for motivational interviewing, 7.08% did not return for follow-up, while only 16% were able to abstain from smoking at the end of the six months. A study undertaken to review the process and operational aspects of the establishment of tobacco cessation clinics (TCC) set up as part of the National Tobacco Control Programme (NTCP) found that 21% had quit smoking at the end of six months; these results were in line with our results [18]. From this study, it was seen that out of 21 students who quit smoking, 76% were those with low nicotine dependence; this again came out to be statistically significant. A study done by Mishra [19] found that people with a higher Fragerstorm score were less likely to quit tobacco which is in consensus with our study. Another study done by Breslau et al. [20] found that smokers with nicotine dependence were 40% less likely to quit in comparison to those who were not dependent; these results are similar to our study. In this study, we tried to find out the number of people who agreed to set a quit date on the entire follow-up visit. A similar study done by Kumar et al. [17] found that 30% had set a quit date at six weeks, while 31% had set a quit date after two months. These findings were not similar to our findings. This might be because we had been motivating our participants intensively with the help of SMS and through a Whatsapp group on a daily basis, which was followed by a counseling session at two weeks and at six months.

In our study, we tried to establish an association between contemplation scores on the three visits. A study done by Ha and Choi [21] found similar results; they showed that the experimental group had a significantly higher stage of change in comparison to the control group. Another interesting finding which came out from our study was the mean number of cigarettes smoked per day. It was seen that the mean number of cigarettes smoked per day was 6.12 on the first visit, and after two weeks, the mean number of cigarettes smoked per day was 4.73. At the six-month follow-up, the mean number of cigarettes smoked came to be 4.08. By applying the ANOVA test, there was an association between the three visits and the number of cigarettes smoked. This was statistically significant (p=0.002). The above findings suggest that there was an overall reduction in the number of cigarettes consumed. In a study done by Jayakrishnan et al. [22], he showed that after six months, 17% had reduced smoking by more than 50%. In our study, we tried to explore the predictors of successful smoking cessation. The findings from our study revealed that the odds of quitting smoking were three times higher among people with a high degree of motivation in comparison to people with a low and moderate degree of motivation; this was statistically significant (p=0.02).

In a study done by Toghianifar et al. [23], 92% had received advice from their family for quitting. They reported that family is the best place to seek for advice; this was in consensus with our study.

It was also observed that the odds of quitting were four times higher among people with a low degree of nicotine dependence in comparison to those with a moderate/high degree of dependence; this was statistically significant (p=0.003). These analyses were in line with a study done by Breslau et al. [20] in 1996, who found that people with low nicotine dependence had 40% more chances of quitting in comparison to those with a high degree of dependence.

In our study, the age at initiation of smoking was not a statistically significant (p=0.406) predictor for smoking cessation, while a study done by Breslau et al. [20] found that the odds of quitting was two times higher times if smoking was initiated after age 17 in comparison to people who started before the age of 17. This difference might have come because, in a study done by Breslau et al. [20], both genders were included, while in our study, only males were included.

The limitation of the study are that female students were not included in the study as the existing social taboo of female smoking behavior in Indian culture might have led to concealment in revealing their true behavior and hence, generated reporting bias. Secondly, smoking status was self-reported. The possibility of fabricated answers could thus not be ruled out. 

## Conclusions

The prevalence of smoking in this present study was in line with the national figures. The study found that the youth most often indulged in smoking for experimental purposes, which in due time transformed into addiction. It was concluded from the study that motivational intervention (MI) is one of the most cost-effective methods in tackling smoking addictions amongst young adults. It was found that with every subsequent session of MI, the determination to quit increases (the mean of contemplation ladder score gradually improved on the three successive follow-up visits). To sum up, MI helped 21% of the young smokers to quit smoking. The association between age of initiation and quitting came out to be highly significant. Smoking is most often started during college life, and this time period during which smoking addiction starts should be targeted for preventive strategies.
